# Vaginal Myomectomy for a Thirteen-Centimeter Anterior Myoma

**DOI:** 10.1155/2013/285243

**Published:** 2013-04-10

**Authors:** Bruno Deval, Pascal Rousset, Salma Kayani

**Affiliations:** ^1^Department of Gynecology, Geoffroy Saint Hilaire Clinic, Paris, France; ^2^Department of Radiology, Paris Descartes University, Public Hospitals of Paris, Hotel Dieu Hospital, Paris, France; ^3^BMI The Somerfield Hospital, Maidstone, Kent ME16 0DU, UK

## Abstract

Vaginal myomectomy is an uncommon but advantageous approach for large interstitial uterine fibroids. Myomectomy is performed via laparotomy and laparoscopy; however, in selected cases, vaginal myomectomy has been proven to be a safe and an effective surgical procedure. We report the case of a 38-year-old para one woman with complaints of chronic lower abdominal pain. Preoperative workup revealed a thirteen-centimeter interstitial uterine myoma in the anterior wall. Successful myomectomy was performed via the vaginal route. We will share the preoperative images, operative technique, and postoperative images.

## 1. Introduction

Uterine fibroids are the most common benign tumours of the female genital tract, affecting 20%–30% of women of reproductive age [1]. This incidence increasse to approximately 70% by age of 49 years [2]. Approximately, 30% of uterine fibroids are associated with symptoms including menorrhagia, pelvic pressure symptoms such as urinary frequency, subfertility, and recurrent pregnancy loss [3]. 

For the removal of symptomatic fibroids, the choices of route include the classical strategy of laparotomy and contemporary strategies including laparoscopy, operative hysteroscopy, or robotic assisted laparoscopic myomectomy. The vaginal route has been underutilized for this procedure. The choice of route for undertaking a myomectomy not only depends upon the size, number, and location of fibroids but also the skill of the surgeon and the facilities available. We report the case of a 38-year-old woman who underwent a myomectomy for a large fibroid via the vaginal route.

First described in 1994 by Magos and colleagues [4] vaginal myomectomy allows surgical management of uterine myomas via vaginal incision through which the myomas are removed and uterine suturing is performed [5]. However this surgical procedure has mainly been applied most of the time for a unique subserosal posterior myoma [5]. Vaginal route is more rarely applicable to anterior myoma [6]. This case report illustrates the feasibility of the vaginal route for large 13 cm anterior myomas.

## 2. Case Presentation

**Figure 1:**
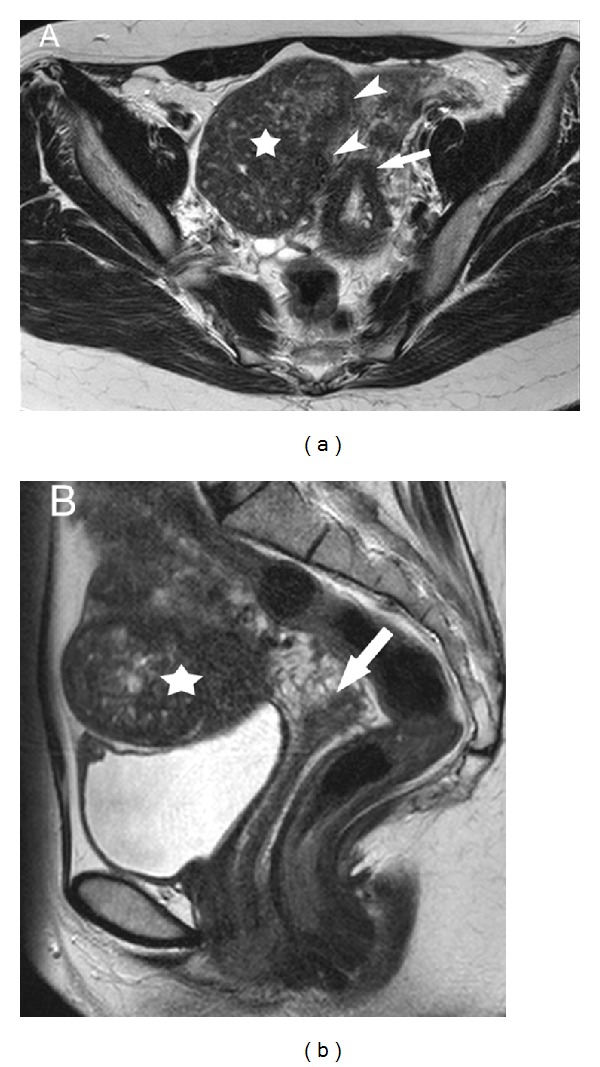
(a) The preoperative axial T2 weighted MR image shows the broad base (arrow heads) of 13 cm myoma (star) laterally and above the isthmus (arrow). (b) The preoperative right parasagittal T2 weighted MR image shows the 13 cm myoma (star) above and anterior to the right vaginal fornix (arrow).

A 38-year-old woman (height 1.61 metres and weight 52 kg) presented with a long history of chronic deep dyspareunia and secondary dysmenorrhea. She was para 1, having had a normal vaginal delivery of a 3.5 kg baby ten years ago. She had no significant medical or surgical history. The clinical examination detected a well-distinguishable mass in the anterior wall of the uterus. The preoperative workup revealed at magnetic resonance imaging (MRI) a large thirteen-centimeter anterior interstitial fibroid (Figures 1(a) and 1(b)). After careful deliberation and multidisciplinary discussions with the radiology team, a decision was taken to undertake the myomectomy via the vaginal route. Patient did not resort to GnRH analogues or preoperative embolisation.

**Figure 2:**
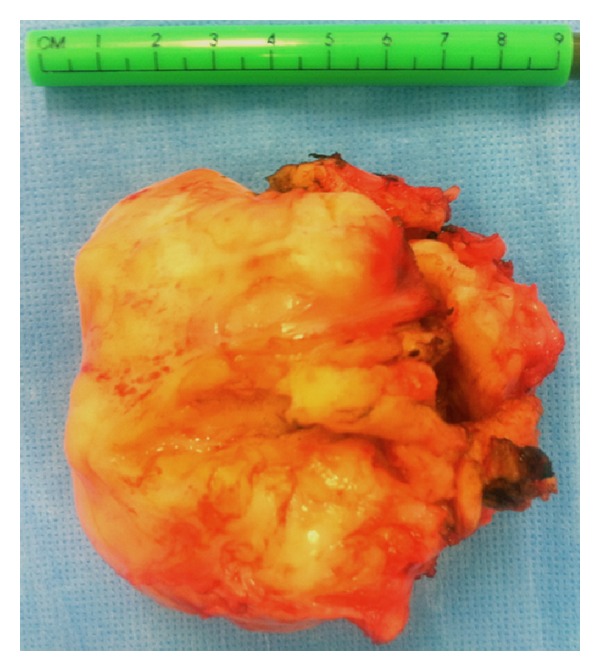
The operative material shows a nine-centimeter large fragment of the myoma.

Surgical procedure was performed under general anesthesia. The uterine mobility through the vagina was preliminary evaluated to ensure that the cervix reached the vulvar vestibulum. Arciform anterior colpotomy was initiated. The myoma was completely enucleated and exteriorized after morcellation via the anterior vaginal colpotomy (Figure 2). Uterine myography was performed in multiple layers with Vicyl 0 to ensure effective hemostasis. Anterior vaginal pouch was closed with separate point Vicryl 0. A vaginal compression and bladder catheterisation were ensured for 24 hours. Operative duration was one hour and blood loss was evaluated at 350 mLs. 

The patient had an uneventful immediate postoperative recovery. Her preoperative haemoglobin was 12 gm/dL and at day 1 postoperative haemoglobin was 11 g/dL. The hospital inpatient stay was 48 hours under antibiotic cover (CEFAZOLINE 2 g/24 H) and Oxytocin (SYNTOCINON) infusion at 15 units/hr for 24 hours. 

**Figure 3:**
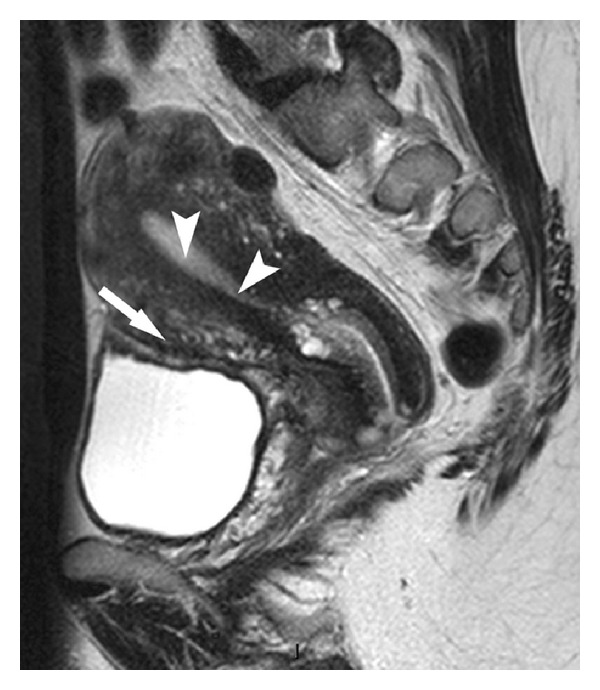
The T2 weighted postoperative pelvic MR image shows no residual myoma. Note the complete uterine cicatrization (arrow) and the regular endometrial cavity with no defect visible (arrowheads).

Clinical examination at follow-up consultation was normal. A postoperative MRI reveals a complete uterine cicatrization (Figure 3). 

## 3. Discussion

The vaginal route is considered the classical way in gynecological surgery. To date, vaginal myomectomy has not been evaluated in prospective cohort studies or randomized controlled trials in comparison to laparotomy, laparoscopic, or robotic assisted laparoscopic myomectomy. Feasibility in well-established situation with specialized team has been described [7]. Adequate vaginal access, uterine mobility, and moderate uterine size are essential prerequisites [7]. Accurate preoperative assessment of myoma's size, localization, and vascularization is the gatekeeper of complication or surgical defeat. 

The size of the myoma could potentially be the reason for abandoning the vaginal route. However, Wei et al. [8] did not find any difference in the weight of the tumor extracted through the vaginal way compared to laparotomy (256 ± 67 (range 88–680) versus 352 ± 86 (range 125–820); *P* = 0.563). In our case, the myoma weight was 580 g. The fibroid was removed by morcellation technique as it was too large to be removed intact. 

Some studies have highlighted the higher risk of abscess or hematoma of the Douglas pouch in case of vaginal access of the pelvic cavity, with a probably underestimated incidence in case of laparotomy [5].

With the increasing use of laparoscopy, our impression is that vaginal route for a myomectomy has been neglected. For myomectomy, vaginal route is a good alternative to laparotomy; however some interstitial myomas may not be readily accessible. Such difficulty could be avoided using a laparoscopic assisted dissection. However exploratory laparoscopy before vaginal myomectomy is not useful to evaluate feasibility of the procedure [5].

Although some authors promote this technique especially for deep posterior myoma, we support and have demonstrated the feasibility and the safety of the vaginal myomectomy even for large anterior myomas. Nevertheless, specific criteria at clinical and imaging examination have to be respected in order to select eligible patient for this surgical strategy. Vaginal surgery allows specific management that must continue to be part of gynaecological surgical training of the future generation of gynecologic surgeons. 

## 4. Conclusion 

Vaginal myomectomy, in well-selected cases, is feasible and well tolerated. However, it should only be undertaken by experienced vaginal surgeons. Size does not seem to be the limitation factor in well-selected cases. Due to the morcellation technique, vaginal myomectomy can be useful even in case of large, numerous, and intramural fibroids and allows optimal uterine wall reconstruction with minimal tissue trauma. The procedure is also less time consuming as compared to laparoscopic myomectomy or robotic assisted laparoscopic myomectomy. This should be incorporated into the curriculum of gynaecological surgical training.
